# Autonomic control of energy balance and glucose homeostasis

**DOI:** 10.1038/s12276-021-00705-9

**Published:** 2022-04-26

**Authors:** Uisu Hyun, Jong-Woo Sohn

**Affiliations:** Department of Biological Sciences, Korea Advanced Institute of Science and Technology, Daejeon, 34141 South Korea

**Keywords:** Autonomic nervous system, Homeostasis

## Abstract

Neurons in the central nervous system (CNS) communicate with peripheral organs largely via the autonomic nervous system (ANS). Through such communications, the sympathetic and parasympathetic efferent divisions of the ANS may affect thermogenesis and blood glucose levels. In contrast, peripheral organs send feedback to the CNS via hormones and autonomic afferent nerves. These humoral and neural feedbacks, as well as neural commands from higher brain centers directly or indirectly shape the metabolic function of autonomic neurons. Notably, recent developments in mouse genetics have enabled more detailed studies of ANS neurons and circuits, which have helped elucidate autonomic control of metabolism. Here, we will summarize the functional organization of the ANS and discuss recent updates on the roles of neural and humoral factors in the regulation of energy balance and glucose homeostasis by the ANS.

## Metabolism: Exploring the influence of the autonomic nervous system

Cutting-edge techniques should be harnessed to unravel how metabolism is modulated by a key part of the body’s nervous system. The autonomic nervous system (ANS) regulates many involuntary physiological processes, such as heart rate, breathing, and blood pressure. Scientists now believe that the ANS is involved in regulating metabolism, but its precise roles are unclear. Jong-Woo Sohn and Uisu Hyun at the Korea Advanced Institute of Science and Technology, Daejeon, Korea, reviewed understanding of how the ANS regulates energy balance, appetite, and glucose homeostasis. Recently-developed mouse models have provided insights into how ANS neurons translate neuronal and hormonal signals into commands during feeding, sending instructions to the liver, and mediating blood glucose levels. Several hormones have been identified that may act on a specific part of the ANS to influence appetite and metabolism.

## Introduction

The autonomic nervous system (ANS) serves as a key structure to mediate unconscious regulation of bodily function by the central nervous system (CNS). In particular, the hypothalamus utilizes the sympathetic and parasympathetic divisions of the ANS to innervate peripheral organs and to control the metabolic function of our body. For instance, anorexigenic (appetite-suppressing) pro-opiomelanocortin (POMC) neurons in the arcuate nucleus of the hypothalamus (ARH) activate sympathetic preganglionic neurons in the spinal cord, which in turn increases thermogenesis in brown adipose tissue (BAT)^1,2^. In addition, POMC neurons in the ARH reportedly regulate parasympathetic preganglionic neurons in the brainstem, which decreases insulin secretion from pancreatic β-cells^3,4^. In these examples, the activity of autonomic neurons is affected by α-melanocyte-stimulating hormone (α-MSH), a neuropeptide released from POMC neurons in the ARH, which acts on the anorectic melanocortin-4 receptor (MC4R). As such, autonomic neurons are influenced by neuropeptides and neurotransmitters^5–8^. In addition, peripheral hormones such as insulin and glucagon-like peptide-1 (GLP-1) were demonstrated to regulate the activity of autonomic neurons^9–11^. Thus, it appears that neurons of the ANS translate neural and humoral signals into commands that directly regulate peripheral organs and metabolic function. Together, these results demonstrate how the efferent (motor) divisions of the ANS regulate metabolism.

However, recent evidence has demonstrated that parasympathetic or vagal afferent (sensory) fibers inform the CNS of food in the gut. For instance, it was demonstrated that ingestion of food causes mechanical stretch of the stomach or intestinal walls, which is relayed by vagal sensory neurons to the CNS and stimulates anorexigenic neurons or inhibits orexigenic (appetite-promoting) neurons to stop feeding^12,13^. In addition, recent studies have suggested that the gut microbiome stimulates vagal sensory neurons to affect many facets of metabolic function^14,15^. Therefore, both efferent and afferent divisions of the ANS can regulate energy balance and glucose homeostasis.

Here, we discuss key structures of the ANS, focusing on the role of ANS neurons in the regulation of feeding and metabolism. We also summarize how neural, humoral, and other factors modulate efferent and afferent divisions of the ANS and their metabolic function.

## Metabolic function of the autonomic nervous system

### Role of autonomic motor function

**Fig. 1:**
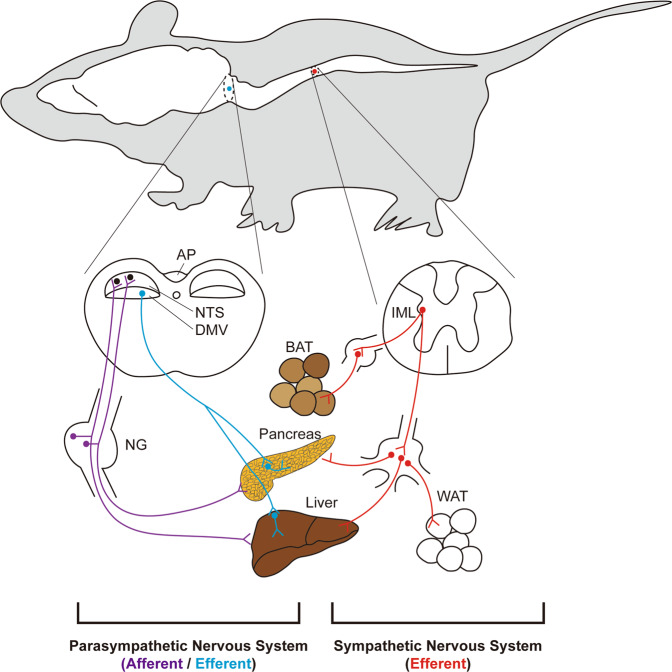
Autonomic innervation of peripheral organs. The parasympathetic preganglionic neurons (blue dots) are located in the DMV of the brainstem, while the sympathetic preganglionic neurons (red dots) are located in the IML of the thoracic and upper lumbar spinal cord. The parasympathetic preganglionic neurons located in the nucleus ambiguus and the IML of the sacral spinal cord are not shown. The parasympathetic postganglionic neurons (blue dots) are located in the peripheral target organs, while the sympathetic postganglionic neurons (red dots) are located in the sympathetic ganglia within the abdominal cavity. The parasympathetic efferent (blue lines) and sympathetic efferent (red lines) fibers innervate peripheral organs that regulate metabolism, including BAT, pancreas, liver, and WAT. Note that BAT and WAT receive only sympathetic innervation, whereas the pancreas and liver are innervated by both parasympathetic and sympathetic efferent nerves. The parasympathetic afferent fibers (purple lines) have cell bodies (purple dots) in the NG, which send peripheral information to neurons of the NTS (black dots) and AP (neurons not shown). See the text for abbreviations.

The parasympathetic and sympathetic nervous systems represent the motor (efferent) part of the ANS, which innervates internal organs and regulates their function^16^ (Fig. 1). Neurons of the ANS, parasympathetic or sympathetic, are categorized into preganglionic and postganglionic neurons; the cell bodies of preganglionic neurons are located within the CNS (brainstem and spinal cord), whereas those of postganglionic neurons comprise autonomic ganglia found in body cavities or peripheral target organs. In particular, the parasympathetic and sympathetic divisions are anatomically segregated. The cell bodies of parasympathetic preganglionic neurons projecting to the internal organs are located in the brainstem, where they make up the dorsal motor nucleus of the vagus (DMV) and the nucleus ambiguus. Notably, although the intermediolateral column (IML) of the sacral spinal cord is conventionally thought to be a part of the parasympathetic division, a recent study suggested that sacral autonomic outflow may be sympathetic^17^ since the developmental and transcriptional traits of sacral autonomic neurons are similar to those of sympathetic preganglionic neurons rather than parasympathetic preganglionic neurons. The parasympathetic postganglionic neurons are located in the target organs and compose synapses with preganglionic axon terminals^8,18^. However, the preganglionic neurons of the sympathetic division are found in the IML of the thoracic to the upper lumbar spinal cord^19,20^. The sympathetic ganglia are typically located outside of the target organs, where the sympathetic postganglionic neurons receive synaptic inputs from the sympathetic preganglionic neurons. For instance, the sympathetic postganglionic neurons innervating the BAT are located in the stellate ganglia^21^, whereas those innervating abdominal organs such as the digestive tract, pancreas, liver, and some white adipose tissues (WAT) are located in the celiac ganglia^22–24^.

Both preganglionic and postganglionic neurons of the parasympathetic division release acetylcholine (ACh) from their terminals. Sympathetic preganglionic neurons also release ACh, but sympathetic postganglionic neurons are unique in that they use norepinephrine (NE) as the major neurotransmitter. Therefore, choline acetyltransferase (ChAT), which is a key enzyme for the synthesis of ACh, can serve as a useful chemical marker for cholinergic autonomic neurons. Using mice with Cre recombinase activity under the control of the ChAT promoter (ChAT-cre mice), researchers manipulated gene expression in a cholinergic neuron-specific manner to identify the role of specific molecules expressed by autonomic neurons^25,26^. Paired-like homeobox 2b (Phox2b) is a transcription factor that is known to mediate the development of the parasympathetic nervous system. Thus, scientists have used Phox2b-cre mice to manipulate neurons of the parasympathetic division of the ANS^26^. No mouse model is currently available to selectively label sympathetic neurons.

The ANS innervates multiple organs that regulate metabolism; the pancreas and the liver receive both sympathetic and parasympathetic innervation, whereas adipose tissues receive only sympathetic innervation^23^ (Fig. 1). The parasympathetic nervous system promotes insulin secretion, as evidenced by the impaired insulin secretion observed in vagotomized rats^27^. However, the sympathetic nervous system stimulates glucagon secretion^28^. A recent study reported that parasympathetic and sympathetic neuronal signaling regulates β-cell proliferation^29^. The parasympathetic and sympathetic nervous systems also affect liver function^18^. The parasympathetic nervous system inhibits the gluconeogenic pathway in the liver^30^, which may contribute to lower blood glucose levels. In contrast, the sympathetic nervous system stimulates gluconeogenic and glycogenolytic pathways in the liver to elevate the blood glucose level^31^. The ANS also has an impact on hepatic lipid metabolism^32^. The sympathetic nervous system enhances very-low-density lipoprotein (VLDL) synthesis and triglyceride (TG) secretion; impaired sympathetic function has been linked to the pathogenesis of nonalcoholic fatty liver disease (NAFLD)^33,34^. In addition, the sympathetic nervous system is an important regulator of WAT function, as evidenced by lipolysis induced by NE released from the synaptic end of sympathetic postganglionic neurons^35^. A recent study reported that sympathetic stimulations even lead to the browning of WAT^36^. The sympathetic nervous system also stimulates thermogenesis in the BAT of rodents^37^. In human subjects, BAT was originally known to exist only in infants, but a recent study reported that adults also have functional BAT^38^. These results further highlight the importance of the sympathetic nervous system as a potential target for the treatment of obesity.

### Role of autonomic sensory function

The sympathetic sensory fibers are intermingled with somatic sensory fibers and thus are not readily dissected anatomically^39^. However, the parasympathetic nervous system has afferent fibers dedicated to sensory function. The vagal sensory neurons are bipolar neurons that project from peripheral organs to the brain stem. The somata of the vagal sensory neurons are located in the nodose ganglia (NG) (Fig. 1). The stimulation of vagal sensory (or NG) neurons was reported to result in the suppression of feeding^40^. Interestingly, experimental evidence from multiple recent studies suggested that NG neurons are highly heterogeneous. A recent study using novel sequencing techniques revealed that NG neurons have a highly localized and compartmentalized structure; the peripheral axons of calcitonin gene-related peptide (CGRP)-expressing NG neurons form a structure called the mucosal endings in the gut, while those of oxytocin receptor (Oxtr)-expressing NG neurons form a structure called the intestinal intraganglionic laminar endings^12^. Notably, optogenetic and chemogenetic activation of Oxtr-expressing NG neurons inhibited food intake, while stimulation of CGRP-expressing NG neurons had no effects. These results suggested that stimulation of a specific subpopulation of NG neurons is sufficient to inhibit feeding. Additionally, it is worthwhile to note that the right and left NGs were reported to be anatomically and functionally distinct^41^. Most neurons in the right NG innervate the nucleus tractus solitarius (NTS), whereas most neurons in the left NG innervate the area postrema (AP). Optogenetic stimulation of axon terminals of NG neurons, right or left, induced a significant decrease in chow intake. However, only stimulation of the right NG neuronal axon terminal resulted in place preference. These results suggested that the activity of the right NG to the NTS circuit is sufficient to induce motivated behavior. The right NG to NTS circuit was found to be connected to the dorsolateral aspect of the parabrachial nucleus (PBN), dopaminergic neurons in the midbrain, and striatum. Finally, a subpopulation of NG neurons was reported to be glucose-sensing neurons^42^. These glucose-sensing neurons may also suppress feeding in vivo, although this hypothesis needs to be confirmed by direct experimental evidence. The vagal sensory neurons, especially those responsible for chemical sensing, can be labeled using Na_v_1.8-cre mice^43^. This mouse model was used to identify the metabolic function of molecules expressed by vagal sensory neurons^44,45^.

As mentioned previously, vagal sensory information is transferred to neurons in the NTS^8^ and AP^41^. The NTS, like the NG, contains many types of neurons that are also functionally heterogeneous. NTS neurons that express either cholecystokinin (CCK) or dopamine β-hydroxylase are activated by food intake, and these neurons provide excitatory input to anorexigenic CGRP-expressing PBN neurons^46^. CCK-expressing NTS neurons were also shown to project to the paraventricular nucleus of the hypothalamus (PVH), which is a major satiety center^47^. In addition, many POMC neurons in the NTS express CCK and the serotonin 2C receptor, which innervate forebrain structures to induce anorexia^47,48^. However, NTS neurons are not always anorexigenic. Tyrosine hydroxylase (TH)-expressing NTS neurons reportedly use NE as a neurotransmitter to innervate orexigenic agouti-related peptide (AgRP) neurons within the ARH, where the release of NE directly excites AgRP neurons^49^. Inhibition of these neurons suppressed food intake when the mice were under glucoprivic hunger, which was induced by 2-deoxyglucose. Interestingly, inhibition of TH-expressing NTS neurons failed to suppress food intake when the mice were subjected to food deprivation.

The PBN receives ascending sensory inputs from the NTS and AP^12^. Recently, a study demonstrated that prodynorphin (Pdyn)-expressing PBN neurons receive information regarding mechanical stretching via the NTS, which mediates anorexia and negative valence^13^. These results suggested that mechanical stretch induced by food in the stomach is sensed by local vagal afferent neurons and transmitted to the NTS and PBN to suppress feeding. Previous studies have suggested that vagus nerve stimulation (VNS), which was originally approved for treatment-resistant epilepsy^50^ and depression^51^, is also effective in reducing food intake^52–54^. VNS is now approved by the Food and Drug Admistration (FDA) for the treatment of obesity. Since VNS affects both the afferent and efferent arms of the vagus nerve through the application of electricity via patches attached to the skin, it is not clear how VNS can reduce food intake. Nonetheless, we envision that the central pathways involving neurons of the NTS, PBN, and possibly the hypothalamus are responsible for the anorexigenic effects.

## Neural control of autonomic function

**Fig. 2:**
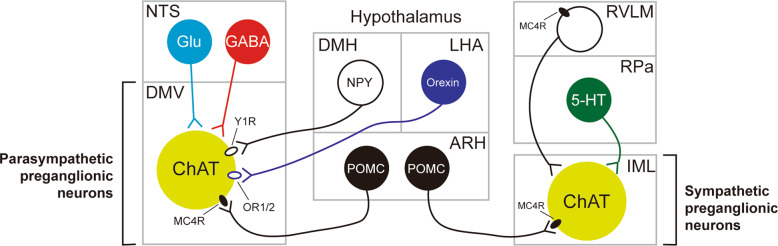
Central neurons that regulate autonomic preganglionic neurons. The parasympathetic preganglionic neurons of the DMV (lower left) receive neural input from neurons of the NTS (upper left) and the hypothalamic nuclei (center). The sympathetic preganglionic neurons of the IML (lower right) receive neural input from neurons of the brainstem (upper right) and ARH POMC neurons (lower center). Only selective major innervations are shown for clarity. See the text for abbreviations.

Parasympathetic preganglionic neurons were previously shown to receive direct and indirect neuronal projections from several nuclei of the hypothalamus^55^ (Fig. 2). Notably, axon terminals that innervate the parasympathetic preganglionic neurons of the DMV are frequently found in the NTS^56^. It is reasonable to assume that neurons in the NTS receive those inputs and relay the information to the parasympathetic preganglionic neurons of the DMV. Indeed, neurons in the NTS directly innervate neurons in the DMV via either GABAergic or glutamatergic fibers^57–59^, and the NTS to DMV GABAergic circuit has been shown to control glucose homeostasis^59^. Alternatively, axon terminals may synapse onto the dendrites of parasympathetic preganglionic neurons that extend into the NTS to directly receive hypothalamic inputs. For example, NPY-expressing DMH neurons monosynaptically innervate Y1 receptor (Y1R)-expressing DMV neurons^60^. This neural circuit was not responsible for the regulation of feeding or body weight but was involved in the maintenance of glucose homeostasis by increasing hepatic glucose production (HGP). In contrast, relatively limited data are available on the neural control of sympathetic preganglionic neurons, which is probably due to the technical difficulty of studying neural circuits in the spinal cord. It was previously shown that neurons in the hypothalamus and brainstem innervate the IML^5,61^, but the functional significance of these connections remains to be determined.

One of the well-characterized inputs to autonomic preganglionic neurons within the DMV and IML originates from arcuate POMC neurons^5,62^. As mentioned previously, POMC neurons release α-MSH, which is a full agonist of MC4R^63^. Both parasympathetic and sympathetic preganglionic neurons express functional MC4Rs^61^. MC4Rs expressed by sympathetic preganglionic neurons were shown to increase BAT thermogenesis and blood pressure but decrease HGP^26^. In addition, MC4Rs expressed by parasympathetic preganglionic neurons were suggested to decrease insulin secretion^26^. Interestingly, patch-clamp studies demonstrated that MC4R agonists depolarize (or activate) sympathetic preganglionic neurons while hyperpolarizing (or inhibiting) parasympathetic preganglionic neurons^25^, which suggests that stimulations of MC4Rs increase sympathetic tone. These results at least in part explain the autonomic phenotypes observed in MC4R-deficient mice and human patients with MC4R mutations, including decreased thermogenesis, hyperinsulinemia, and resistance to obesity-induced hypertension. However, it remains unclear how MC4Rs normalize (or reduce) HGP by increasing sympathetic activity. In addition, there is currently no evidence that the activity of autonomic preganglionic neurons is modulated by α-MSH release in vivo. These remaining issues need to be resolved in future investigations. However, little is known regarding the regulation of the ANS by other neuropeptides released from hypothalamic neurons. One example is orexin (or hypocretin), which is a neuropeptide synthesized by a discrete set of neurons within the lateral hypothalamic area (LHA) to control feeding behavior and arousal^64^. It was previously demonstrated that LHA orexin neurons project to gut-projecting DMV neurons that putatively express orexin receptor 1/2 (OR1/2) to control gastric function^65,66^.

Neurons that directly innervate sympathetic preganglionic neurons in the IML are called sympathetic premotor neurons^67^. Sympathetic premotor neurons are typically found in the rostral medulla. Sympathetic premotor neurons within the rostral ventrolateral medulla (RVLM) are known to control cardiovascular functions^68^. In particular, MC4R-expressing RVLM neurons innervate the IML neurons that project to the lung^69^. Unlike the RVLM, those in the rostral medullary raphe regions reportedly regulate thermogenesis^70,71^. In particular, sympathetic premotor neurons located within the rostral part of the raphe pallidus (RPa) and raphe magnus were suggested to be involved in thermoregulation. A previous study demonstrated that optogenetic stimulation of cholinergic neurons decreased BAT thermogenesis via muscarinic M2 receptors expressed by RPa serotonergic neurons^72^. In another study, serotonergic neurons located in the dorsal raphe nuclei (DRN) projected to the RPa and functionally modulated BAT energy expenditure^6^. Given the role of DRN serotonergic neurons in regulating thermogenesis and locomotor activity^73,74^, the DRN→RPa circuit may represent an effector system to excite the sympathetic nervous system.

## Humoral control of autonomic function

Parasympathetic preganglionic neurons are also influenced by peripheral hormones, which may enter the CNS via circumventricular organs (CVOs) where the blood-brain barrier is not very tight^75,76^. Thus, peripheral hormones may have access to neurons within the NTS and DMV via the AP, which has characteristics of CVO. In the case of sympathetic preganglionic neurons, there is no nearby structure that can serve as a CVO. Therefore, sympathetic preganglionic neurons have only limited access to peripheral hormones, which may be why there are currently no data regarding humoral regulation of sympathetic preganglionic neurons. Most results regarding the role of hormones in the regulation of autonomic neurons were obtained from studies using in vivo conditional knockout mouse models and ex vivo electrophysiology experiments.

Leptin and leptin receptors (LepRs) were first reported in the 1990s^77,78^. Leptin is a unique fat cell-derived hormone, and many scientists have studied this hormone in the context of feeding and metabolism. Indeed, mice and human subjects lacking leptin or LepRs develop obesity, which is accompanied by decreased energy expenditure and increased food intake^79,80^. In particular, the abundant expression of LepRs by central neurons has prompted researchers to study the role of leptin in the CNS^81–84^. While deletions of LepRs in a single population of neurons failed to reproduce the obesity phenotypes observed in whole-body knockout mice^85,86^, Lowell and colleagues found that LepR deficiency in GABAergic neurons produces obesity^87^. These results highlighted the role of GABAergic neurons in mediating the metabolic effects of leptin, but the anatomical location of the responsible GABAergic neurons is still unknown. In the ANS, LepR deficiency in Phox2b neurons did not result in a body weight phenotype, although both food intake and energy expenditure were increased^88^. These results suggest that LepRs expressed by parasympathetic neurons cause changes in either food intake or energy expenditure, which is readily compensated. Multiple studies from independent groups reported that leptin applications inhibit the activity of DMV neurons via phosphoinositide 3-kinase (PI3K)-dependent activation of ATP-sensitive potassium (K_ATP_) channels^89,90^ (Table 1). However, it is currently not clear whether leptin-induced inhibition of DMV neurons causes changes in food intake or energy expenditure. Insulin is another peripheral hormone that controls parasympathetic neurons. It was reported that insulin also inhibits DMV neurons via PI3K-dependent activation of *K*_ATP_ channels^9^. Interestingly, parasympathetic preganglionic neurons stimulate the secretion of insulin from pancreatic β-cells^3^. Therefore, the suppression of DMV neuronal activity by insulin may represent a negative feedback loop. However, it remains to be determined whether such homeostatic regulation exists in animals.

**Table 1:** Hormones that regulate parasympathetic neurons.

Investigated brain area	Hormone	Effects on neuronal activity	Proposed mechanism	Ref.
DMV	Leptin	Hyperpolarization	↑ K^+^ conductance	^89^
		Hyperpolarization	↑ *K*_ATP_ conductance (PI3K-dependent)	^90^
	Insulin	Hyperpolarization	↑ *K*_ATP_ conductance (PI3K-dependent)	^9^
	GLP-1	Depolarization	↓ K^+^ conductance (direct) and/or ↓ Cl^−^ conductance (indirect)	^92^
	CCK	Generation of inward current	↓ K^+^ conductance	^94^
		Depolarization	↓ K^+^ conductance (direct) ↑ sEPSC frequency (indirect)	^95^
NTS	CCK	Activation (↑ c-Fos)	N. A.	^97^
		N. A.	↑ pERK1/2	^98^

In addition to leptin and insulin, hormones released from gut endocrine cells were demonstrated to affect autonomic function. For instance, while GLP-1 is secreted largely from gut endocrine cells, GLP-1 has its cognate receptor (GLP-1 receptor or GLP-1R) throughout the brain. In particular, it was shown that DMV neurons also express GLP-1R^91^ and that pancreas-projecting GLP-1R-expressing neurons are excited by the application of GLP-1 directly via the closure of putative K^+^ conductance and indirectly via GABA-activated Cl^-^ conductance^92^. The excitation of DMV neurons and the increased parasympathetic tone may contribute to the well-known insulinotropic effects of GLP-1. Interestingly, by using Phox2b-cre-specific GLP-1R knockout mice, researchers reported that the conditional knockout mice show decreased food intake, glucose tolerance, and accelerated gastric emptying^10^. However, knockdown of GLP-1R in neurons of the NTS reportedly resulted in increased food intake in the dark cycle^11^.

Another example is CCK, which was originally identified as a gut modulator acting on vagal afferent fibers^93^. CCK is released from duodenal endocrine cells in isoforms such as CCK58, CCK22, and CCK8. Neurons of the DMV express CCK receptor 1 (CCK1R), and CCK8 generates inward currents by decreasing putative K^+^ conductance^94^. Later, it was shown that CCK8 depolarizes the membrane potential of DMV neurons directly by decreasing K^+^ currents and indirectly by decreasing the frequency of spontaneous excitatory postsynaptic currents (sEPSCs)^95^. CCK1R is also expressed by neurons of the NTS^96^, and it was shown that in the postprandial period, c-Fos activity and phosphorylated extracellular signal-regulated kinase 1/2 (pERK1/2) levels are increased in CCK1R-expressing NTS neurons^97,98^. Moreover, CCK was demonstrated to activate NTS POMC neurons, which may play a role in generating satiety^99,100^. Therefore, both GLP-1 and CCK appear to affect appetite and metabolism by acting on both motor and sensory parts of the parasympathetic nervous system.

## Concluding remarks

The ANS has a major role in the control of energy balance and glucose homeostasis; sympathetic activity increases thermogenesis and hepatic gluconeogenesis, parasympathetic activity promotes insulin secretion, and vagal sensory neurons signal fullness. Therefore, it is essential to determine the mechanisms in autonomic neurons and the circuits to obtain a comprehensive understanding of whole-body metabolism in health and disease. “Conventional” autonomic neuroscience utilizes histology and electrophysiology as the major tools. Currently, findings obtained from these experiments are continuously being corroborated with findings using fine genetic tools, including mouse genetics, optogenetics, and chemogenetics. As a result, we now have more detailed information regarding autonomic control of appetite and metabolism.

Given that neurons of the ANS not only regulate appetite and metabolism but also control a variety of key homeostatic functions, such as cardiac activity and breathing, it is very likely that other functions, including circulation and respiration, influence metabolism and that the ANS serves as an important mediator between these functions. For example, we need more blood and oxygen to metabolize nutrients after each meal, and the ANS likely performs fine-tuning of these homeostatic functions. Fortunately, many advancements have recently been made in other fields of neuroscience, and the cutting-edge techniques used therein could be applied to study autonomic function. However, autonomic circuitry is not as straightforward as central neural circuits since the former includes the interface between peripheral organs and peripheral/central neurons. Therefore, we need to focus on autonomic neuroscience and develop more advanced methods to investigate autonomic function and circuits. We believe that these efforts will help to gain novel insight into the autonomic function and to result in additional therapeutic options for obesity and metabolic diseases.
